# Supplementary material for: Towards unified AI-driven fracture mechanics: the extended deep energy method (XDEM)
Source: Nat Commun. 2026 Aug 15;17:8492. doi: 10.1038/s41467-026-76748-1 (PMC13484471; doi:10.1038/s41467-026-76748-1)
Supplement: Supplementary file 3 [file 41467_2026_76748_MOESM3_ESM.zip › pic/Method/XDEM.pdf]

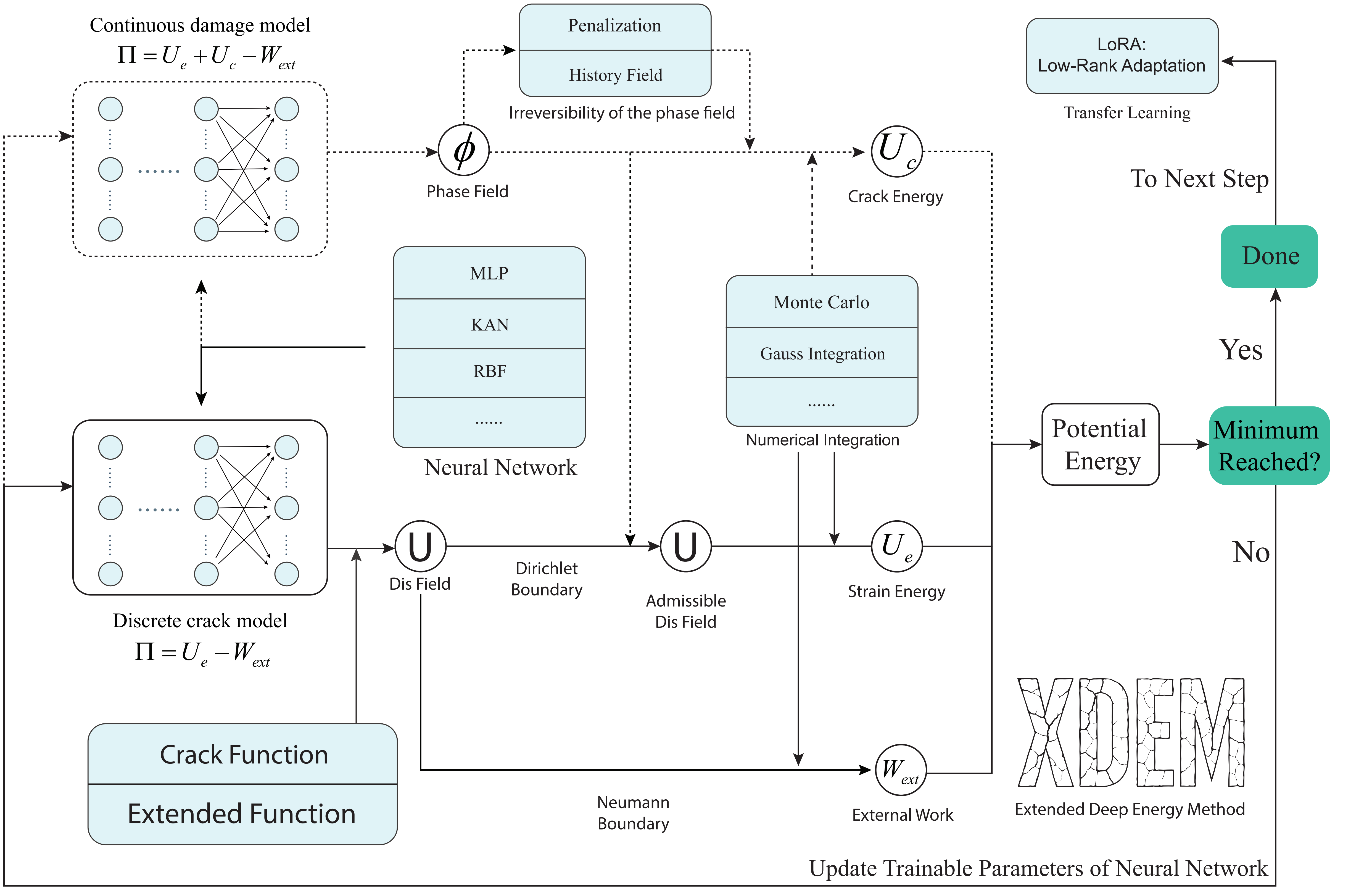

Continuous damage model
$\Pi = U_e + U_c - W_{ext}$
Penalization
History Field
Irreversibility of the phase field
LoRA:
Low-Rank Adaptation
Transfer Learning
ϕ
Phase Field
U_c
Crack Energy
To Next Step
Done
Yes
MLP
KAN
RBF
......
Neural Network
Monte Carlo
Gauss Integration
......
Numerical Integration
Potential Energy
Minimum Reached?
No
U
Dis Field
Dirichlet Boundary
U
Admissible Dis Field
U_e
Strain Energy
Discrete crack model
$\Pi = U_e - W_{ext}$
Crack Function
Extended Function
Neumann Boundary
W_{ext}
External Work
XDEM
Extended Deep Energy Method
Update Trainable Parameters of Neural Network
